# Eutrophication and Warming Boost Cyanobacterial Biomass and Microcystins

**DOI:** 10.3390/toxins9020064

**Published:** 2017-02-11

**Authors:** Miquel Lürling, Frank van Oosterhout, Elisabeth Faassen

**Affiliations:** 1 Aquatic Ecology & Water Quality Management Group, Department of Environmental Sciences, Wageningen University, P.O. Box 47, 6700 AA Wageningen, The Netherlands; jean.vanoosterhout@wur.nl (F.v.O.); els.faassen@wur.nl (E.F.); 2 Department of Aquatic Ecology, Netherlands Institute of Ecology (NIOO-KNAW), P.O. Box 50, 6700 AB, Wageningen, The Netherlands; 3 Environmental Risk Assessment, Wageningen Environmental Research, Wageningen Resesarch, P.O. Box 47, 6700 AA Wageningen, The Netherlands

**Keywords:** cell quota, climate change, cyanobacterial blooms, cyanotoxins, mitigation, seston

## Abstract

Eutrophication and warming are key drivers of cyanobacterial blooms, but their combined effects on microcystin (MC) concentrations are less studied. We tested the hypothesis that warming promotes cyanobacterial abundance in a natural plankton community and that eutrophication enhances cyanobacterial biomass and MC concentrations. We incubated natural seston from a eutrophic pond under normal, high, and extreme temperatures (i.e., 20, 25, and 30 °C) with and without additional nutrients added (eutrophication) mimicking a pulse as could be expected from projected summer storms under climate change. Eutrophication increased algal- and cyanobacterial biomass by 26 and 8 times, respectively, and led to 24 times higher MC concentrations. This effect was augmented with higher temperatures leading to 45 times higher MC concentrations at 25 °C, with 11 times more cyanobacterial chlorophyll-*a* and 25 times more eukaryote algal chlorophyll-*a*. At 30 °C, MC concentrations were 42 times higher, with cyanobacterial chlorophyll-*a* being 17 times and eukaryote algal chlorophyll-*a* being 24 times higher. In contrast, warming alone did not yield more cyanobacteria or MCs, because the in situ community had already depleted the available nutrient pool. MC per potential MC producing cell declined at higher temperatures under nutrient enrichments, which was confirmed by a controlled experiment with two laboratory strains of *Microcystis aeruginosa.* Nevertheless, MC concentrations were much higher at the increased temperature and nutrient treatment than under warming alone due to strongly promoted biomass, lifting N-imitation and promotion of potential MC producers like *Microcystis*. This study exemplifies the vulnerability of eutrophic urban waters to predicted future summer climate change effects that might aggravate cyanobacterial nuisance.

## 1. Introduction

The incidence and intensity of cyanobacterial blooms are on the rise worldwide [1,2,3]. Cultural eutrophication—i.e., the over-enrichment of surface waters with nutrients, primarily nitrogen (N) and phosphorus (P)—is frequently a key driver of cyanobacterial bloom formation [4,5]. In addition, there is a broad consensus that global warming will further promote the worldwide proliferation of cyanobacterial blooms [2,6,7]. Warming and warming-enhanced nutrient loading are predicted to intensify eutrophication symptoms [8] and modeling forecasts suggest that warming-augmented run-off and influx of nutrients will increase phytoplankton biomass with a dominance of potentially toxin-producing cyanobacteria [9]. In those scenarios, run-off is primarily restricted to enhanced average winter precipitation, yet a rise in short intense summer storms during periods of droughts is also expected as a result of climate change [10]. Such events will fuel receiving waters with a pulse of nutrients during the growing season that may further promote cyanobacteria [11].

Cyanobacterial blooms may cause unpleasant sights and odors, create turbid water and may cause fish kills, but the most important reason why cyanobacterial blooms are viewed as problematic is that they may present a serious health threat because of the potent toxins they might produce [12]. Among a variety of cyanobacterial toxins produced, microcystins (MCs) are most frequently encountered in freshwater blooms all around the world [13]. Likewise, in The Netherlands, MCs are by far most abundant and are found at higher concentrations than the other toxins [14,15]. MC concentrations in lakes can vary over orders of magnitude [16,17] and can be strongly related to *Microcystis* abundance [17,18]. 

Cyanobacterial blooms often are comprised of toxic and non-toxic strains, where the toxic ones could benefit more from warming and eutrophication than the non-toxic ones [19]. Although the ample availability of nutrients is a strong promoter of cyanobacterial abundance [5], changes in the relative nutrient availability could also influence MC-cell quota [20] or the relative composition of MC variants produced [17,21]. Controlled experiments with isolated strains seem to indicate that toxin-quotas are also influenced by changing temperatures. For instance, Gianuzzi et al. [22] found a decrease in MC cell quota when *M. aeruginosa* was cultured at 29 °C compared to 26 °C. Brutemark et al. [23] found higher MC cell quota when *Dolichospermum* sp. was cultured at 21 °C compared to 17 °C. This was also observed by Rapala & Sivonen [24], who, however, studied MC content over 10 to 28 °C and clearly observed a sharp drop in MC contents at the highest temperature. Two *Dolichospermum* strains had the highest MC quota at 25 °C [25]. In tropical *Microcystis* species, MC quota at 36 °C were significantly lower than at lower temperatures [26]. Temperature had no significant effect on the MC quota in *M. viridis* [27], while *M. aeruginosa* became less toxic at higher temperatures [28,29] or contained less MCs at 30 °C than at 20 °C [30]. Hence, it seems that generally MC quota might drop at elevated temperatures though these studies have been conducted primarily with laboratory strains. Therefore, we decided to expose natural seston from a eutrophic urban pond to different temperatures with and without nutrient addition, mimicking a pulse under various climate scenarios: cold weather, normal summer, and extreme summer. We tested the hypothesis that warming promotes cyanobacteria more than algae and that eutrophication enhances cyanobacterial biomass and MC concentrations.

## 2. Results

### 2.1. Chlorophyll-a Concentrations and Cell Concentrations in Incubated Natural Seston

**Figure 1:**
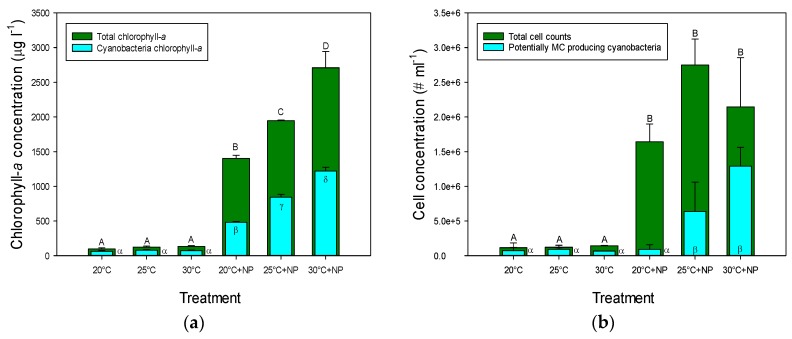
(**a**) Cyanobacterial- and total chlorophyll-*a* concentrations (μg·L^−1^) in incubations of water samples from an urban pond incubated for one week at three different temperatures without and with addition of NaNO_3_ and K_2_HPO_4_ (+NP) to mimic warming and eutrophication; (**b**) Total phytoplankton cell concentrations (cells·mL^−1^) and cell concentrations for potential microcystin (MC) producing species. Error bars indicate 1 SD (*n* = 3), while different symbols (A,...,D; α,...,δ) indicate groups that are statistically different (Tukey test; *p* < 0.05).

There was a clear response of adding nutrients (14 mg·N·L^−1^ as NaNO_3_ and 1.4 mg·P·L^−1^ as K_2_HPO_4_), on the total- and cyanobacterial chlorophyll-*a* concentrations (determined with a PHYTO-PAM) as well as on phytoplankton cell concentrations (Figure 1). Adding nutrients boosted phytoplankton biomass, both in terms of chlorophyll-*a* concentrations (Figure 1a) and in terms of cell concentrations (Figure 1b). A two-way ANOVA on log-transformed total chlorophyll-*a* concentrations indicated a significant temperature effect (*F*_2,17_ = 36.0; *p* < 0.001), a significant eutrophication effect (*F*_1,17_ = 1935; *p* < 0.001) and a significant temperature x eutrophication interaction effect (*F*_2,17_ = 15.8; *p* < 0.001). A temperature effect was only found when nutrients were added; a Tukey test revealed that, under eutrophic conditions, total chlorophyll-*a* concentrations significantly increased with increasing temperature (Figure 1a). Similarly, a two-way ANOVA on cyanobacterial chlorophyll-*a* concentrations indicated a significant temperature effect (*F*_2,17_ = 231.0; *p* < 0.001), a significant eutrophication effect (*F*_1,17_ = 2993; *p* < 0.001) and a significant temperature x eutrophication interaction effect (*F*_2,17_ = 217.9; *p* < 0.001). Cyanobacterial chlorophyll-*a* concentrations were similar in all three temperature treatments when no nutrients were added, but were significantly higher than the treatments without nutrient addition and significantly different from each other at each temperature when nutrients were added (Figure 1a). 

Cell concentrations showed a pattern more or less comparable with that of chlorophyll-*a* with much higher cell numbers when nutrients were added (Figure 1b). For total cell concentrations (log-transformed data), however, a two-way ANOVA indicated no temperature effect (*F*_2,17_ = 2.11; *p* = 0.164) and no temperature x eutrophication interaction (*F*_2,17_ = 0.88; *p* = 0.441), but only a significant eutrophication effect (*F*_1,17_ = 380.5; *p* < 0.001). Zooming in on the different phytoplankton organisms revealed some differences in responses to temperature and/or nutrient enrichment (Appendix A). Colonies of very small-celled cyanobacteria (here referred to as pico-cyanobacteria) increased between 11 and 148 times when water was enriched with nutrients, *Microcystis* sp. increased between 2 and 27 times and *Dolichospermum* sp. between 2 and 17 times. Also, cell counts of chlorophytes (14–18 times) and diatoms (4–10 times) were higher under nutrient enrichments. In contrast, crytophytes were no longer observed under enrichment, *Aphanizomenon flos-aquae* disappeared in enriched treatments at 25 °C and 30 °C, while *Woronichinia naegeliana* was no longer observed in the 30 °C + NP treatments (Appendix A). The share of diazotrophs in the cyanobacteria communities remained similar around 6%; a two-way ANOVA on square root transformed proportions revealed no eutrophication effect (*F*_1,17_ = 0.91; *p* = 0.360), no temperature effect (*F*_2,17_ = 0.01; *p* = 0.989), and no temperature x eutrophication interaction (*F*_2,17_ = 0.18; *p* = 0.830) (Appendix A).

Potentially MC producing cyanobacteria (i.e., *Dolichospermum, Microcystis, Woronichinia*) were dominated by *Microcystis* sp. and likewise followed the same pattern to enrichment and warming (Appendix A). A two-way ANOVA on the potentially MC producers specified a significant temperature effect (*F*_2,17_ = 10.9; *p* = 0.002), a significant eutrophication effect (*F*_1,17_ = 32.1; *p* < 0.001) and a significant temperature x eutrophication interaction effect (*F*_2,17_ = 8.92; *p* = 0.004). Tukey test revealed two groups: (1) the temperature treatments (20, 25, 30 °C) together with the 20 °C plus nutrients added (20 °C + NP); and (2) the two elevated temperatures with nutrients added (25 °C + NP, 30 °C + NP) (Figure 1b).

### 2.2. Growth Rates of Incubated Natural Seston

The estimated cyanobacterial chlorophyll-*a* based growth rates were in all incubations higher than the estimated algal chlorophyll-*a* based growth rates (Table 1). A two-way ANOVA on cyanobacterial growth rates indicated a significant temperature effect (*F*_2,17_ = 36.0; *p* < 0.001), a significant eutrophication effect (*F*_1,17_ = 11935; *p* < 0.001), and a significant temperature x eutrophication interaction effect (*F*_2,17_ = 15.8; *p* < 0.001). In the series without nutrient addition, cyanobacterial growth rates were significantly higher in 25 °C treatments compared to the 20 °C incubations, while they were significantly different in each of the enriched incubations (Table 1).

### 2.3. Microcystins in Incubated Seston

**Figure 2:**
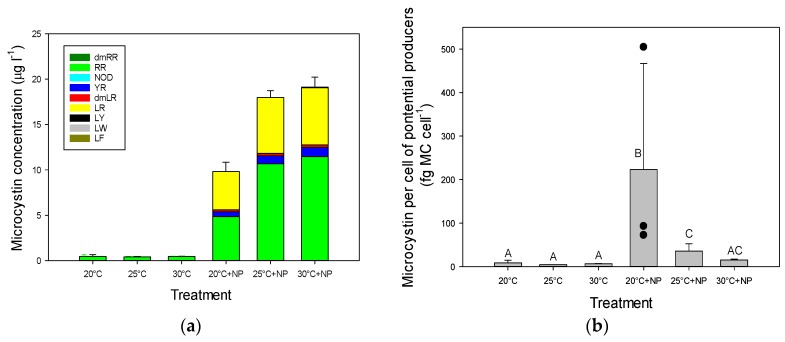
(**a**) Concentrations of different microcystin (MC) variants (μg·L^−1^) in water samples from an urban pond that had been incubated for one week at three different temperatures without and with addition (+NP) of NaNO_3_ (14 mg N·L^−1^) and K_2_HPO_4_ (1.4 mg P·L^−1^) to mimic warming and eutrophication; (**b**) Total MC per cell (fg MC·cell^−1^) for potentially microcystin (MC) producing species. Error bars indicate 1 SD (*n* = 3), while different symbols (A,...,C) indicate groups that are statistically different (Tukey test; *p* < 0.05). Black circles in the 20 °C + NP treatment are the individual replicates.

Adding nutrients also increased MC concentrations (Figure 2a). A two-way ANOVA on log-transformed total MC concentrations indicated a significant temperature effect (*F*_2,17_ = 5.99; *p* = 0.016), a significant eutrophication effect (*F*_1,17_ = 1643; *p* < 0.001), and a significant temperature x eutrophication interaction effect (*F*_2,17_ = 6.28; *p* = 0.014). Tukey’s test disclosed that MCs were similar in the three treatments without nutrient addition, significantly elevated in the 20 °C plus nutrients treatment (20 °C + NP) and highest in the two highest temperatures when nutrients were also added (25 °C + NP, 30 °C + NP) (Figure 2a). In all treatments, MC-RR was most abundant. The variants MC-YR and MC-LR were detected in all incubations without nutrient addition, but were below the corresponding level of detection (LOD). The variant dmMC-LR was detected twice. When nutrients were added, these three variants could be quantified, whereas the variant MC-LY was around the level of detection. One sample contained a trace of dmMC-RR (Figure 2a).

A two-way ANOVA on log-transformed total MC per cell of potentially MC producing cyanobacteria found a significant temperature effect (*F*_2,17_ = 6.13; *p* = 0.015), a significant eutrophication effect (*F*_1,17_ = 43.7; *p* < 0.001), and a significant temperature x eutrophication interaction effect (*F*_2,17_ = 5.08; *p* = 0.025). A Tukey post-hoc comparison test revealed that MC per cell of potentially MC producing cyanobacteria in the 20 °C + NP and 25 °C + NP were significantly higher than those in the treatments without nutrient addition (Figure 2b). When MC concentrations were expressed per unit cyanobacterial chlorophyll-*a*, this pattern was less clear. The two-way ANOVA showed no temperature effect (*F*_2,17_ = 2.81; *p* = 0.100), a significant eutrophication effect (*F*_1,17_ = 122.9; *p* < 0.001), and no temperature x eutrophication interaction (*F*_2,17_ = 2.99; *p* = 0.088). However, when only the nutrient enriched treatments were compared, the one-way ANOVA indicated significant differences (*F*_2,8_ = 9.34; *p* = 0.014) and the MC:chlorophyll-*a* ratio in the 30 °C + NP treatment was significantly lower than in the 20 °C +NP and 25 °C +NP treatments (Table 2).

### 2.4. Growth Rates, Chlorophyll-a, and Microcystins in M. Aeruginosa Strains

Given the tendency to lower MC:chlorophyll-*a* concentrations at higher temperatures under nutrient replete conditions and that it is impossible to quantify the chlorophyll-*a* of only the MC producing cyanobacteria, we performed an additional experiment with two *M. aeruginosa* strains. 

The growth rates of the two *M. aeruginosa* strains were significantly different from each other as indicated by a two-way ANOVA (*F*_1,24_ = 347; *p* < 0.001). Also, temperature had a significant effect (*F*_5,24_ = 448; *p* < 0.001) on the chlorophyll-*a* based growth rates and this effect was different in both strains as indicated by a significant temperature x strain interaction effect (*F*_5,24_ = 21.9; *p* < 0.001). Tukey’s test revealed that, at each temperature, the growth rate in populations of strain PCC7941 were significantly higher than those of the NIVA-CYA140 strain at the corresponding temperature. The optimum growth rate was determined at 30 °C in both strains (Table 3). These two strains produced primarily dmMC-LR and MC-LR. When cultured in a temperature range between 20 °C and 35 °C significant differences between total MC concentrations and MC to chlorophyll-*a* ratios were found (Table 3). In strain PCC7941, the MC to chlorophyll-*a* ratios dropped from 0.128 μg·MC·μg^−1^ chlorophyll-*a* at 20 °C to 0.001 at 35 °C, and in the NIVA-CYA140 strain it dropped from 0.328 at 20 °C to 0.002 at 35 °C.

## 3. Discussion

The results of our study support the hypothesis that warming directly promotes cyanobacterial growth, as growth rates were 24% and 15% higher at 25 °C and 30 °C than at 20 °C. A direct warming effect on growth rates has been proposed as one of the mechanisms giving cyanobacteria a competitive advantage over their eukaryote competitors at elevated temperatures [6,7]. Controlled experiments, however, showed that optimum growth temperatures and growth rates at the optimum temperature were similar for cyanobacteria and chlorophytes [31]. Our data also reveal that, at the lowest temperature, cyanobacterial growth rates by far exceeded algal growth rates. One explanation could be that the low N conditions of the pond water (see Appendix C) favored diazotrophs, and not eukaryote algae. Nonetheless, the share of diazotrophs in the cyanobacterial community was rather low, only a few%, and did not change when the pond water was enriched with nutrients. Inasmuch as the flasks were continuously shaken, enhanced sedimentation of non-cyanobacterial species is not a likely explanation for higher cyanobacterial growth rates than algal growth rates. More likely, and seemingly supported by negative growth rates (indicating losses), is that the algae suffered more from grazing by a zooplankton community of small cladocerans, rotifers, and a few copepodites than the cyanobacteria [32,33]. Cyanobacterial biomass was, although not statistically significant, 30% and 18% higher at 25 °C and 30 °C, respectively, than at 20 °C. 

Adding nutrients multiplied cyanobacterial growth rates and also stimulated algal growth. Consequently, phytoplankton biomass increased under these nutrient enrichments. It has been widely recognized that eutrophication and warming are the main drivers of cyanobacterial blooms [2,6,8] and that warming may exacerbate cyanobacterial nuisance [6,8]. Our study clearly supports that warming and eutrophication may act in synergy [7] as after a pulse of nutrients simply increasing the temperature from 20 °C to 25 °C yielded 39% more chlorophyll-*a* and an increase to 30 °C gave a 93% increase. For cyanobacterial chlorophyll-*a* this was even 174% and 252% at 25 °C and 30 °C, respectively. Hence, when a summer storm has brought in nutrients, a subsequent warm period can then easily facilitate a dense cyanobacterial bloom. Such short intense summer storms during periods of droughts are predicted to occur more frequently as a result of climate change [10]. In fact, the typical heat wave in 2006 already showed an extreme summer precipitation event in a very dry and warm period in The Netherlands [34]. That event caused a major change in the Molenwiel pond where the water was taken from in this study. Despite there was already a bloom preceding the summer storm, which was dominated by *Aphanizomenon flos-aquae* and an undergrowth of *Woronichinia naegeliana* and some *Microcystis*, the summer storm and influx of run-off water with nutrients caused a short peak in diatoms that was rapidly replaced predominantly by *Microcystis* and *Woronichinia* accumulating in thick scums on the water surface. The chlorophyll-*a* concentrations in these surface accumulations ranged from 5 to 130 mg·L^−1^, while MC concentrations in these surface accumulations had increased on average from 100 μg·MC·L^−1^ before to 4700 μg·MC·L^−1^ after the summer storm.

The augmented nutrient influxes and higher temperatures could not only potentially increase the frequency, biomass, duration, and distribution of cyanobacterial blooms [2,35], but also their toxicity [2,19]. In general, higher temperatures lead to a larger proportion of toxic *Microcystis* cells in field populations, whereas higher temperatures coupled with elevated phosphorus concentrations promote growth rates of toxic *Microcystis* cells [19]. Some studies found a correlation between intracellular MCs and the percentage of toxic *Microcystis* cells [36], suggesting that a greater percentage of toxic *Microcystis* cells may imply that warming and eutrophication could lead to cyanobacterial blooms with greater MC concentrations [19,37,38]. Surface water MC concentrations, however, do not only depend on the cyanobacterial biomass and the amount of toxic cells, but also on MC cell quotas [39]. MC cell quotas are not only determined by the presence of the synthesizing genes, but also by environmental factors that influence MC synthesis or MC fate [39]. Although our results with pond water cannot underpin that temperature has an effect on MC quota as no quantification of MC-producing cells was made, we used the potential MC producers as a proxy instead. When total MC was expressed per potential MC producing cell, a decline with warmer temperatures was observed, particularly under nutrient enriched conditions. The large standard deviation was caused by a relatively low number of potentially toxic MC cells in one of the replicates, but leaving this one replicate out clearly showed that also in the remaining two replicates the MC per potential MC producing cell was much larger than at warmer temperatures (see Figure 2b). Given that others found a stimulation of toxic cells under warming and eutrophication [19], we assume toxic cells were not drastically reduced at warmer temperatures in our experiment with pond water and thus that MC per potential MC producing cell dropped because of lower MC synthesis, higher degradation, or higher association with proteins. This would then imply a lower MC quota at higher temperatures, which seems supported by our experiment using two toxic *M. aeruginosa* strains that obviously contained less MC at higher temperatures. Likewise, several studies have reported lower MC cell quotas at higher temperatures [22,24,25,26,30]. The laboratory experiment with the two *M. aeruginosa* strains was a batch experiment where, due to growing population size, nitrogen could have become limited, which may have led to lower MC contents [40]. While in the study of Van de Waal et al. [40] 10 days in N-deplete conditions caused a drop in MC content to about 30% of the initial content, the reduction in the two *M. aeruginosa* strains in our experiment was much stronger as, in the two highest temperatures, the MC to chlorophyll-*a* ratio was reduced to around 1% of the initial ratios. These populations still expressed exponential growth, cyanobacteria contain cyanophycin that serves as nitrogen storage [40] and given the close relationship between chlorophyll cell quota and MC cell quota in *M. aeruginosa* [41] a direct temperature effect seems more likely than N-limitation.

There was a discrepancy between the cyanobacterial cell counts in the 20 °C + NP treatment and the measured cyanobacterial chlorophyll-*a* concentrations and the MC concentrations, which was most probably caused by an underestimation of the actual number of cells in the relatively large *Microcystis* colonies. This will have influenced the MC to potential MC producing cell ratio determination. However, given that our controlled experiment with two *M. aeruginosa* strains gave comparable results it does not affect our conclusion of probable lower MC cell quota at higher temperatures, which is further supported by numerous other studies as outlined above.

Recent studies have elucidated that a large portion of MCs is covalently bound to proteins [42,43,44]. Standard hot extraction will not liberate these bound MCs, and thus this pool has been overlooked using our standard analysis based on aqueous methanol extractable MCs. High light conditions have been identified as a steering factor in promoting the protein-associated MC pool ([43,44]), yet in our experiment light was not so high that it may have caused oxidative stress. A faster growth under warmer conditions could have generated these cells in a late exponential phase which is associated with higher oxidative stress and lower MC-quota due to a potentially higher protein-bound fraction [42]. Some conjugates, like MC-LR-glutathione and MC-LR cysteine conjugates expressed only 6% and 14% of the toxicity of unconjugated MC-LR [45] and thus bound MCs may be less toxic. It remains to be seen, however, if these bound MCs are easily freed. Based on the rather irreversible thioether bond between the Mdha methylene group of MC and the thiol group of cysteine, Meissner et al. [43] indicated that there is no reason for water authorities to abandon the established analytical protocols for the detection of MCs.

Regardless of whether or not lower MC cell quotas are a result of shifting proportions between a non-bound and bound pool, nutrient enrichment and warming caused an overwhelming biomass effect, where extractable MC concentrations were 83% and 95% higher at 25 °C and 30 °C, respectively than at 20 °C. In contrast, in non-nutrient enriched conditions, MC concentrations remained similar despite the higher temperatures. Since the pond water was low in dissolved nitrogen (Appendix C), N-limitation might have caused relatively low MC-concentrations. The elevated MC to chlorophyll-*a* ratio under enriched conditions points towards relatively enhanced MC synthesis, which is a common phenomenon following a nitrogen pulse [40]. A clear difference in the MC concentrations between the experiment with pond water and with the two *M. aeruginosa* strains was observed. In the pond water experiment, nutrient addition and warming yielded higher MC concentrations, while warming led to lower MC concentrations in the experiment with the two strains. This apparent discrepancy is most probably caused by a combination of lifting N-limitation in the pond water, a relatively large biomass effect, and a potential stimulation of MC producers—in particular *Microcystis* sp. (Appendix A). A similar phenomenon was observed in the pond in 2006 where, after a summer storm, the phytoplankton community shifted to dominance of *Microcystis* sp. 

Warming, however, also intensifies stratification and lengthens the period of stratification, therewith favoring the development of surface accumulations [7]. Thus, although MC concentrations remained similar in our continuous mixing experiment when only the temperature of water from a eutrophic pond was increased, in situ aggravated accumulation of cyanobacteria could lead to much higher cyanobacterial and MC concentrations locally than predicted from our experiment, representing a high risk of adverse health effects [46]. In comparison, the nutrient effect was much stronger than the warmer effect: at 20 °C eutrophication caused, on average, 24 times higher MC concentrations, at 25 °C it was 45 times higher, and at 30 °C 42 times.

**Figure A2:**
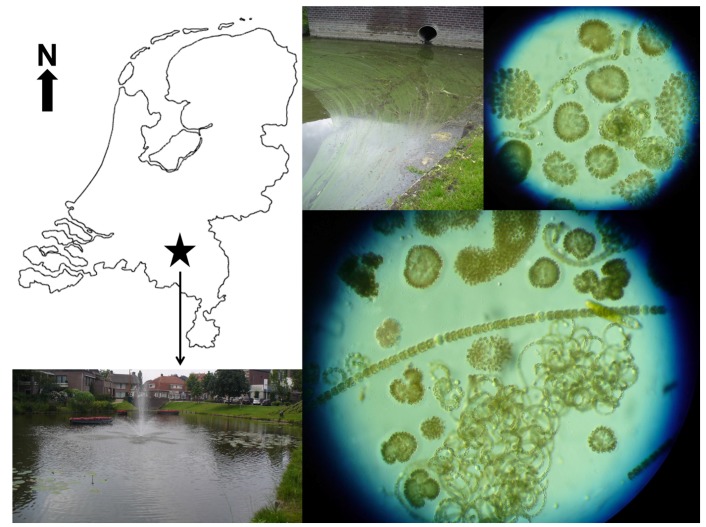
Location of pond Molenwiel in The Netherlands (★), as well as pictures of the pond on 29 July, 2011 (left below), a minor surface accumulation and the main phytoplankton species in this accumulation.

Our results unambiguously show that warming and eutrophication act in synergy in stimulating algal and cyanobacterial biomass, but also that eutrophication is the most dominant factor, which is in line with a large dataset analysis on the relative importance of nutrients and temperature in steering cyanobacterial biomass [47]. The role of grazing zooplankton in facilitating cyanobacterial blooms should not be underestimated [32,33], which was indicated from losses measured for algal chlorophyll-*a*. The water used in the experiment originated from a eutrophic urban pond, but a clear interaction between trophic status and temperature only appeared when additional nutrients were added. This is quite logical as the growing biomass had reduced the available nutrient pool to low levels and the community seemingly had gone into an N-limitation as the molar DIN:Phosphate-P ratio of 3 (Appendix C) was below 13 [48]. It is straightforward that in this urban pond additional adding of N (and P) will cause more algal- and cyanobacterial biomass as was evidenced in our experiment. Impeding any additional inflow will prevent it, yet such inflow control is rather difficult to realize given summer storm heavy precipitation pulses. Cyanobacteria were already accumulating at the water surface in this pond (Figure A2) meaning that in order to lessen blooms, available resources should be lowered prior to summer storm nutrient pulses. Low-nutrient waters are more resilient to a pulse of nutrients, because of a completely different plankton community and ecosystem structure. In addition, low-nutrient lakes, ponds, and reservoirs will mostly not build blooms under warmer conditions and thus they are more resilient to the expected adverse effects of warming [49]. Warming is rather difficult to tackle for lake managers, but measures to reduce the nutrient load and trophic state of a water body are more feasible [49]. Therefore, catchment and in situ measures to curb cyanobacterial nuisance are strongly needed in a warming world.

## 4. Conclusions

A pulse of nutrients to natural seston from a eutrophic urban pond boosted algal- and cyanobacterial biomass. This effect was augmented with warmer temperatures, but warming alone did not yield more cyanobacteria or MCs, because the in situ community had already depleted the available nutrient pool. Despite the fact that MC per potential MC producing cell declined at higher temperatures under nutrient enrichments, MC concentrations were highest under these conditions, due to strongly promoted biomass, lifting N-limitation, and stimulation of potential MC producers (*Microcystis* sp.). To mitigate the expected aggravation of cyanobacterial blooms as a result of pulsed summer precipitation and warmer periods, the nutrient status of water bodies should be drastically lowered.

## 5. Materials and Methods

### 5.1. Sampling

On 29 July, 2011, we sampled the urban pond Molenwiel (51°33′57.57′′ N; 5°27′44.60′′ E) located in the village Sint-Oedenrode in the Netherlands (Appendix C). Dissolved oxygen concentration and saturation (OxyGuard Handy Polaris, OxyGuard International A/S, Farum, Denmark), conductivity (WTW-Cond 330i, WTW GmbH & Co. KG, Weilheim, Germany), pH (WTW-pH320), water temperature and Secchi-depth were measured on site (Appendix C). A sampling tube was used to collect four liters of pond water. In the laboratory total- and cyanobacterial chlorophyll-*a* concentrations were measured using a PHYTO-PAM phytoplankton analyzer (Heinz Walz GmbH, Effeltrich, Germany). Turbidity was measured with a Hach 2100P Turbidity meter (Hach Nederland, Tiel, The Netherlands). Unfiltered samples were analyzed on total phosphorus (TP) concentration using a Skalar SAN++ Segmented Flow Analyzer (Skalar Analytical B.V., Breda, The Netherlands) following the Dutch standard protocols [50]. Glass-fiber filtered (Whatman GF/C, Whatman International Ltd., Maidstone, UK) samples were analyzed on dissolved inorganic nitrogen (DIN, i.e. ammonium and nitrate plus nitrite) and phosphate concentrations (Skalar SAN++ Segmented Flow Analyzer; [50,51,52]). A qualitative microscopic inspection was performed using a Nikon light microscope (phytoplankton) and a stereo-binocular microscope (zooplankton) (Nikon Instruments Europe BV, Amsterdam, The Netherlands).

### 5.2. Experiment Pond Water

Water from the pond was used in an experiment to test the effect of warming and/or nutrient addition on phytoplankton growth, final chlorophyll-*a* concentrations, composition of major phytoplankton groups—i.e., cyanobacteria and eukaryote algae–and MC concentrations. Eighteen 100 mL Erlenmeyer flasks were filled with 50 mL pond water. Nine flasks were enriched with nutrients; nitrogen (14 mg·N·L^−1^ as NaNO_3_) and phosphorus (1.4 mg·P·L^−1^ as K_2_HPO_4_), while nine received no additional nutrients. The flasks were closed with cellulose plugs. Three flasks with nutrient addition and three without nutrient addition were incubated for one week in a Sanyo Gallenkamp (SANYO Electric Co., Ltd., Osaka, Japan) incubator at 20 °C, three other flasks with and three without nutrients added were placed in an incubator at 25 °C, while the remaining six flasks were incubated at 30 °C (Appendix D). These temperatures were based on water temperatures measured during several summer field campaigns from 2006 to 2011 that revealed 20 °C as a common average summer water temperature, 25 °C as a typical warm summer temperature, while 30 °C was included as an extreme summer temperature. In all three incubators, light was provided by fluorescent tubes that illuminated the flasks from above at 140 µmol photons m^−2^·s^−1^. Light was provided in 18:6 h light:dark cycles and flasks were shaken continuously at 75 rpm.

Initially and after one week incubation, cyanobacterial and eukaryote algae chlorophyll-*a* concentrations were measured using the PHYTO-PAM. The PHYTO-PAM uses four different excitation wavelengths, which allows a separation between cyanobacteria, green algae, and diatoms/dinoflagellates, but also detects other eukaryote phytoplankton in the water. We refer to chlorophyll-*a* concentrations determined in the blue channel as cyanobacterial chlorophyll-*a* and the sum of the green and brown channel as eukaryote algae chlorophyll-*a*. Total chlorophyll-*a* is the sum of all three channels. We used the chlorophyll-*a* concentration as an endpoint, because in general chlorophyll-*a* is considered a reliable measure of the response to eutrophication [53]. After one week of incubation, cell counts were performed microscopically using a Sedgwick Rafter counting chamber, where cyanobacteria were identified to the genus or species if possible, and algae were classified in major groups (chlorophytes, diatoms, cryptophytes, and other). Aliquots of 30 mL were filtered over a glass-fiber filter (Whatman GF/C) and stored at −20 °C for MC analysis. 

### 5.3. Experiment with Two Microcystis Aeruginosa Strains

Two *M. aeruginosa* (Kützing) Kützing 1846 strains (NIVA-CYA140 and PCC7941) were maintained at 22 °C in 250 mL Erlenmeyer flasks and kept on modified WC (Woods Hole modified CHU10)-medium [54]. The cultures were illuminated in a 16:8 h light:dark cycle at 80 μmol quanta m^−2^·s^−1^. Prior to the experiment, aliquots of the stock cultures were acclimatized to the experimental conditions in separate incubators (Sanyo, MLR-351H, SANYO Electric Co., Ltd., Osaka, Japan) at six different temperatures, which were either 20, 25, 27.5, 30, 32.5, or 35 °C. The experiment was started by transferring inocula to 100 mL medium in clean 250 mL Erlenmeyer flasks such that the initial cyanobacteria concentration in each flask was 50 μg chlorophyll-*a*·L^−1^. The flasks were placed for four to six days in the incubators and were shaken manually two times a day. Chlorophyll-*a* concentrations were measured daily using the PHYTO-PAM. After four or six days, 30 mL of each flask was filtered over a glass-fiber filter (GF/C, Whatman International Ltd., Maidstone, UK) and stored at −20 °C for MC analysis.

### 5.4. Microcystin (MC) Analysis

Samples were analyzed for eight MC variants (dm-7-MC-RR, MC-RR, MC-YR, dm-7-MC-LR, MC-LR, MC-LY, MC-LW, and MC-LF) and nodularin (NOD) by LC-MS/MS as described in [45,46]. In short, LC-MS/MS analysis was performed on an Agilent 1200 LC and an Agilent 6410A QQQ (Agilent Technologies, Santa Clara, CA, USA). MCs were separated on an Agilent Eclipse XDB-C18 4.6 × 150 mm, 5 μm column. Calibration standards were obtained from DHI LAB Products (Hørsholm, Denmark). Detailed information on extraction, elution program, and MS/MS settings for each MC can be found in [16]; information on recovery, repeatability, limit of detection, and limit of quantification of the analysis is given in [55].

### 5.5. Data Analysis

In the pond water experiment, cyanobacterial- and total chlorophyll-*a* concentrations, cell counts, total MC concentrations, and MC cell quota were statistically compared by running separate two-way ANOVAs with temperature and nutrient addition (yes/no) as the fixed factors in the program SigmaPlot (version 13.0; Systat Software Inc., San Jose, CA, USA). Data were tested for normality (Shapiro-Wilk) and equal variance (Brown-Forsythe). If the normality test failed, data were log-transformed to fulfil the requirements for ANOVA. A Tukey post hoc comparison test was used to distinguish significantly different means.

Chlorophyll-*a* based growth rates were calculated for both cyanobacteria and algae using the separation obtained by the PHYTO-PAM. Assuming exponential growth over the seven day incubation period, growth rates (μ, d^−1^) were determined from the difference in chlorophyll-*a* concentrations at the start of the incubation and after seven days according to:

$$\mu =\frac{\left(\ln\left(CHL_{end}\right)-\ln(CHL_{start}\right))}{7}$$

Growth rates were initially analyzed by three-way ANOVA with temperature, nutrient addition (yes/no), and cyanobacteria/algae as fixed factors. Given that the ANOVA indicated a statistically significant interaction between temperature, nutrients, and cyanobacteria/algae, which means that the effect of one factor is not consistent at all combinations of the two other factors (making an unambiguous interpretation of the main effects impossible), separate t-tests were performed on some of the main comparisons. A two-way ANOVA was run on cyanobacterial growth rates with temperature and nutrient addition (yes/no) as the fixed factors, followed by a Tukey’s test to distinguish differences.

In the experiment with *M. aeruginosa* strains, growth rates were calculated as in the assay with natural seston. The course of the chlorophyll-*a* concentrations confirmed that cultures expressed exponential growth (Appendix B). MC concentrations were analyzed per strain with incubation temperature as the fixed factor using a one-way ANOVA (NIVA-CYA140) or a Kruskal-Wallis One Way Analysis of Variance on Ranks (PCC7941), because the normality test failed.

## Figures and Tables

**Table 1:** Mean growth rates (μ ± 1 SD, d^−1^) of cyanobacteria and algae based on chlorophyll-*a* concentrations, including their pairwise comparisons. Different letters (A,..., E) for cyanobacterial growth rates indicate homogeneous groups that are significantly different (Tukey test; *p* < 0.05).

Growth Rates (d^−1^)			
Treatment	Cyanobacteria	Algae	Pairwise Comparison
20 °C	0.16 ± 0.03 ^A^	−0.04 ± 0.02	*t*_4_ = 10.0; *p* < 0.001
25 °C	0.20 ± 0.02 ^B^	−0.01 ± 0.03	*t*_4_ = 10.0; *p* < 0.001
30 °C	0.19 ± 0.02 ^AB^	0.04 ± 0.01	*T*_3_ = 10.0; *p* = 0.100 ^#^
20 °C + NP	0.46 ± 0.01 ^C^	0.43 ± 0.01	*t*_4_ = 8.04; *p* = 0.001
25 °C + NP	0.54 ± 0.01 ^D^	0.45 ± 0.01	*t*_4_ = 16.8; *p* < 0.001
30 °C + NP	0.59 ± 0.01 ^E^	0.50 ± 0.02	*t*_4_ = 9.03; *p* < 0.001

^#^ = Mann-Whitney Rank Sum Test, because normality test failed.

**Table 2:** Total microcystin (MC) to cyanobacterial chlorophyll-*a* ratios (means ± 1 SD in μg·μg^−1^; *n* = 3). Different letters (A,B) indicate homogeneous groups (Tukey test; *p* < 0.05).

Treatment	MC:Chlorophyll
20 °C	0.008 ± 0.005 ^A^
25 °C	0.005 ± 0.002 ^A^
30 °C	0.006 ± 0.001 ^A^
20 °C + NP	0.020 ± 0.003 ^B^
25 °C + NP	0.021 ± 0.001 ^B^
30 °C + NP	0.016 ± 0.001 ^B^

**Table 3:** Growth rates (μ, d^−1^) over a four day period, final chlorophyll-*a* concentrations (μg·L^−1^) and total MC concentrations (μg·L^−1^) of two *Microcystis aeruginosa* strains grown for four or six days at different temperatures (Appendix B). ^#^ are data measured after six days. Data given are means ± 1 SD (*n* = 3). ND = not determined. Different letters (A,...,E) per column indicate homogeneous groups that are significantly different (Tukey test; *p* < 0.05).

	Growth Rate (d^−1^)		Chlorophyll-*a* (μg·L^−1^)		Total MC (μg·L^−1^)	
Treatment	PCC7941	CYA140	PCC7941	CYA140	PCC7941	CYA140
20 °C	0.52 ± 0.02 ^A^	0.24 ± 0.06 ^A^	1404 ± 197 ^#^	133 ± 30	178 ± 7.1 ^A #^	43 ± 11 ^A^
25 °C	0.74 ± 0.01 ^B^	0.69 ± 0.03 ^B^	2635 ± 1188 ^#^	803 ± 82	49 ± 7.6 ^AB #^	79 ± 19 ^B^
27.5 °C	0.94 ± 0.02 ^CE^	0.71 ± 0.01 ^B^	2156 ± 153	859 ± 41	36 ± 2.5 ^AB^	36 ± 3.1 ^A^
30 °C	1.01 ± 0.02 ^D^	0.94 ± 0.01 ^C^	2905 ± 262	2186 ± 107	ND	16 ± 1.2 ^C^
32.5 °C	0.99 ± 0.01 ^CD^	0.88 ± 0.03 ^D^	2603 ± 46	1704 ± 181	3.6 ± 2.4 ^B^	5.9 ± 0.6 ^D^
35 °C	0.90 ± 0.02 ^E^	0.71 ± 0.02 ^B^	1842 ± 179	871 ± 71	2.6 ± 0.6 ^B^	1.6 ± 0.4 ^E^

**Figure A1:**
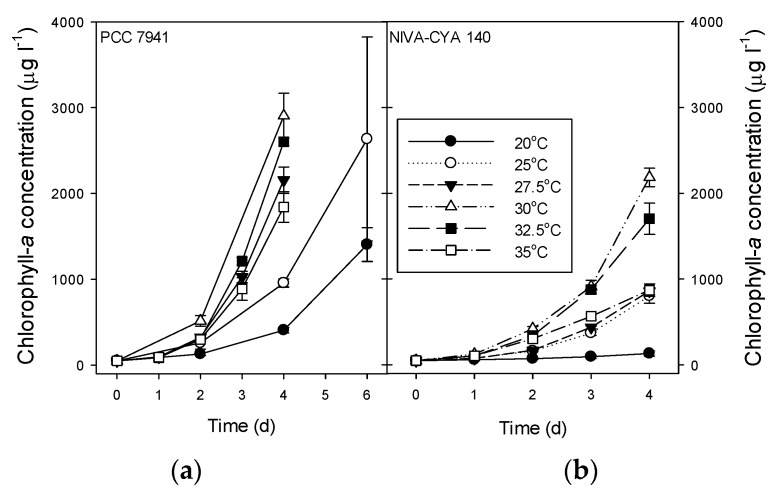
(**a**) Course of the chlorophyll-*a* concentrations (μg·L^−1^) of *Microcystis aeruginosa* PCC7941 cultured at six different temperatures (20–35 °C); (**b**) Course of the chlorophyll-*a* concentrations (μg·L^−1^) of *Ms aeruginosa* NIVA-CYA140 cultured at six different temperatures (20–35 °C); Error bars indicate 1 SD (*n* = 3).

**Figure A3:**
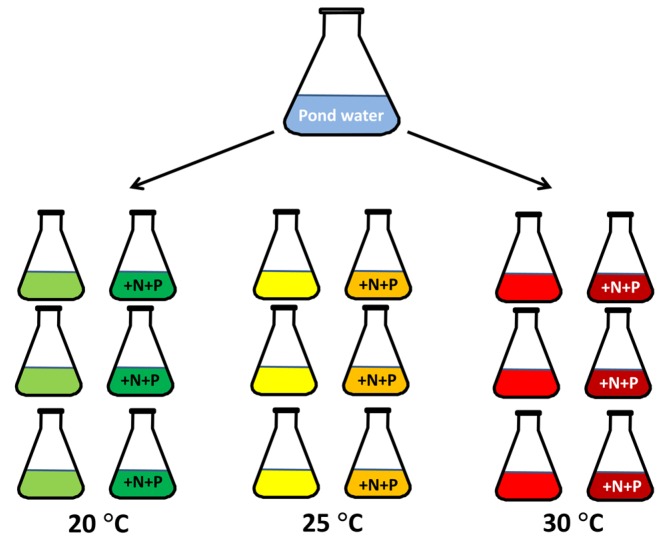
Experimental design in which water from the urban pond Molenwiel was enriched with nutrients (+N + P) or not enriched and incubated for one week at three different temperatures.

## Appendix A

Cell counts using a Sedgwick Rafter counting chamber of samples taken after one week incubation of pond Molenwiel water at three temperatures. The values are means of three replicates with the standard deviation between brackets. Letters per row (A, B) indicate homogeneous groups for those phytoplankton of which the data met the requirements for two-way ANOVA (Table A1).

**Table A1:** Cell counts (mean with 1SD in parantheses; *n* = 3) of samples taken after one week incubation of pond Molenwiel water at three temperatures (20 °C, 25 °C, 30 °C) without and with nutrients added (+N +P). – indicates no cells were detected. Similar letters in a row indicate homogeneous groups (Tukey’s test).

	20 °C	25 °C	30 °C	20 °C	25 °C	30 °C
Organisms				+N + P	+N + P	+N + P
Pico-cyanobacteria	29,212 ^A^ (22,948)	13,000 ^A^ (10,476)	51,167 ^A^ (6371)	1,323,333 ^B^ (304,737)	1,921,667 ^B^ (319,074)	571,667 ^AB^ (770,460)
*Aphnanizomenon flos-aquae*	1964 (1418)	3200 (3464)	2400 (2078)	42,000 (72,746)	-	-
*Dolichospermum* sp.	1667 (2082)	2000 (1732)	1000(-)	3333 (5774)	20,000 (10,000)	16,667 (11,547)
*Microcystis* sp.	41,439 ^A^ (45,991)	48,000 ^A^ (24,434)	47,000 ^A^ (8352)	74,167 ^A^ (68,298)	498,333 ^B^ (205,933)	1,275,000 ^B^ (277,804)
*Woronichinia naegeliana*	28,364 (630)	41,333 (2309)	18,667 (10,066)	13,333 (23,094)	120,000 (207,846)	-
Chlorophytes	10,030 ^A^ (4371)	12,867 ^A^ (3523)	16,467 ^A^ (1361)	181,333 ^B^ (35,388)	180,000 ^B^ (68,440)	258,667 ^B^ (43,143)
Diatoms	755(342)	967 (611)	5367 (1756)	3333 (4163)	9667 (10,017)	22,000 (15,716)
Cryptophytes	648 (89)	400 (265)	333 (252)	-	-	-
Others	315 (223)	33 (58)	-	-	-	-
Potential MC producing	71,470 (47,698)	91,333 (24,214)	66,667 (3014)	90,833 (67,376)	638,333 (421,733)	1,291,667 (270,386)
Total cyanobacteria cells	102,645 (65,134)	107,533 (32,305)	120,233 (5301)	1,456,167 (292,218)	2,560,000 (377,227)	1,863,333 (762,534)
Diazotrophs	3630 (2220)	5200 (4583)	3400 (2078)	45,333 (70,038)	20,000 (10,000)	16,667 (11,547)
Total cells	114,394 (68,915)	121,800 (28,574)	142,400 (6075)	1,640,833 (255,225)	2,749,667 (373,305)	2,144,000 (710,762)

## Appendix B

The course of the chlorophyll-*a* concentrations of the two *Microcystis aeruginosa* strains (NIVA-CYA140 and PCC7941) that were cultured at six different temperatures during four or six days (Figure A1).

## Appendix C

The urban pond Molenwiel in the village Sint-Oedenrode (The Netherlands, 51°33′57.57′′ N; 5°27′44.60′′ E) was sampled on 29 July, 2011 (Figure A2).

The main water quality variables determined on 29 July, 2011 in pond Molenwiel were:

Temperature:	19.5 °C
pH:	8.13
Conductivity:	392 μS·cm^−1^
O_2_ concentration:	9.1 mg·L^−1^
O_2_ saturation:	96%
Turbidity:	28.5 NTU
Secchi depth:	40 cm
Cyano CHL-a:	19.6 μg·L^−1^
Total CHL-a:	65.5μg·L^−1^
Total-P:	76 μg·P·L^−1^
Phosphate-P:	5.6 μg·SRP·L^−1^
DIN:	36 μg·N·L^−1^

## Appendix D

The design used in the experiment in which water from the urban pond Molenwiel was transferred into 100 mL Erlenmeyer flasks that were either enriched with nutrients (+N + P; 14 mg·N·L^−1^ as NaNO_3_ and 1.4 mg·P·L^−1^ as K_2_HPO_4_) or received no additional nutrients and that were incubated for seven days at 20, 25, or 30 °C (Figure A3).
